# Microbiome harbored within tumors: a new chance to revisit our understanding of cancer pathogenesis and treatment

**DOI:** 10.1038/s41392-020-00244-1

**Published:** 2020-07-29

**Authors:** Kunming Zhao, Ying Hu

**Affiliations:** 1 Shenzhen Graduate School of Harbin Institute of Technology, Shenzhen, 518055 China; 2 School of Life Science and Technology, Harbin Institute of Technology, Harbin, 150001 HL China

**Keywords:** Cancer microenvironment, Cancer

**In a recent study in*Science*, Nejmen et al. provide the first comprehensive characterization of the tumor microbiome. Their data reveal that the microbiome mostly resides intracellularly in both tumor and immune cells, and that its composition varies with tumor type and subtype, smoking status, and immunotherapy response.**

**Fig. 1:**
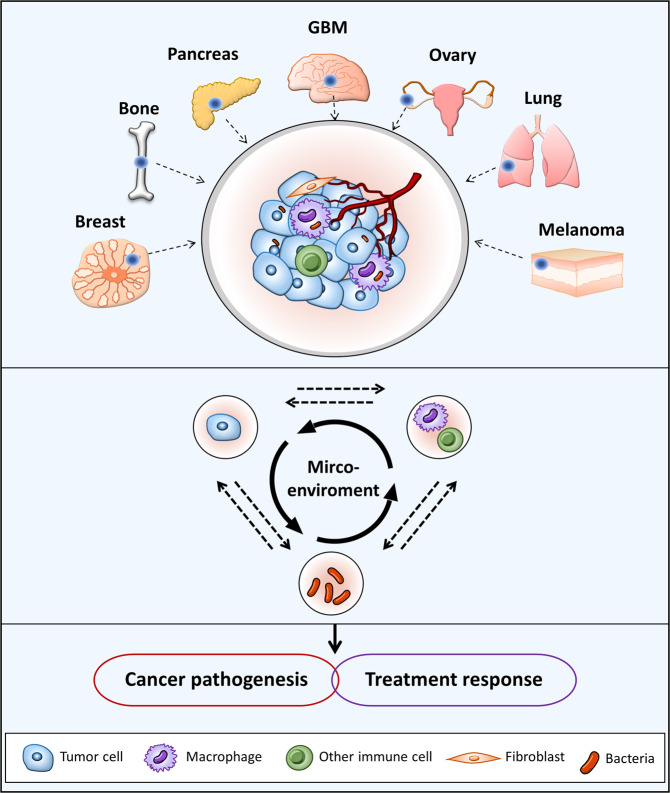
Microbiome in tumors may be actively involved in microenvironment reconstruction and then play roles in the cancer pathogenesis and treatment response

Mounting evidence suggests the active involvement of the tumor microenvironment in pathogenesis and treatment responses,^1^ leading to the newly accepted concept that tumors are more like holistic intricate biosystems than a collection of malignant cancer cells.^2^ Innate and adaptive immune cells, blood and lymphatic vessel networks, and stromal cells are general components that constitute the tumor microenvironment. Recently, an increasing number of tumors have been reported to harbor bacteria, raising the possibility that the microbiome may be an additional player in the complex tumor ecosystem. Nonetheless, due to their relatively low biomass and the suspicion of sample contamination, both the existence and function of the tumor microbiome remain issues under debate. In a recent publication in *Science*, Nejmen et al.^1^ accomplished, for the first time, a comprehensive analysis of the tumor microbiome, examining 1526 samples and corresponding normal adjacent tissue samples across seven types of tumors, including both common tumor types (breast, lung, ovary, and pancreatic) and others with little known microbiome information (melanoma, bone, and brain) using a single platform. To reduce the potential effects of contamination before and during analysis, the authors set up 437 DNA extraction controls, 206 PCR template-free controls, and 168 paraffin controls. In addition, they combined an improved 16S ribosomal RNA gene-sequencing method with microscopy and culture-based techniques to characterize tumor-residing bacteria in a more precise manner. By doing so, they found that each tumor type had a distinct microbiome composition, despite a varied bacteria detection rate ranging from 14.3% in melanoma to >60% in breast, pancreatic, and bone tumors. Remarkably, bacteria were found to be predominately intracellular and to reside in both tumor and immune cells. They further validated the associations between specific bacteria, tumor types and subtypes, smoking history, and sensitivity to immunotherapy, highlighting a potential function of the microbiome in tumor behavior and treatment responses. These important findings raise diverse issues that warrant further investigation (Fig. 1).

First, the fact that microbiomes are commonly harbored within tumors leads to an interesting question about what makes up the tumor microbiome. With advances in molecular and culture techniques, many tissues that have been traditionally considered sterile have been shown to harbor low-abundance microbial communities. The data from Nejmen et al.^1^ also reveal that the microbial compositions of different tumors are similar to those of their normal adjacent tissues. Thus, it is possible that tumor microbiomes evolve from bacteria that exist in normal tissues. Alternatively, the occurrence of tumor microbiomes may result from the systemic chronic inflammation, local vascular permeability, and immunosuppressive or metabolic status within tumors.^3^ Furthermore, in some types of tumors (i.e. pancreatic), the microbiome may be initiated by bacterial translocation from the intestinal tract. This model is supported by the observation that both pancreatic cancer and colon tissues harbor similar bacterial taxa.^1^ These bacteria may then be subjected to further selection by the distinctive pathological characteristics of the tumor, resulting in a relatively high bacterial load and temporally stable tumor-specific microbiome features.

Second, despite a significant association between the tumor microbiome and some pathological features of tumors, direct evidence regarding the effect of the tumor microbiome on the development of cancer is lacking. Microorganisms have been suspected to play a role in carcinogenesis for a long time. For example, the bacterium *Helicobacter pylori* has been classified as a human carcinogen that induces chronic inflammation in human gastric mucosa and gradually promotes the development of stomach cancer. Although preclinical and clinical studies suggest that, in most cases, microbially driven carcinogenesis is caused by a global change to the microbiome and not of a single pathogen, it is plausible that specific bacteria or microbiome compositions can influence the natural history of a tumor by modulating the host’s immune response. In line with this idea, Riquelme and colleagues have successfully modulated the pancreatic tumor microbiome, which subsequently elicited a robust effect on tumor growth and tumor immune infiltration.^4^ In addition, emerging evidence has also shown an active role of bacteria in the development or progression and metastasis of malignancies by inducing chronic inflammation, genotoxicity, angiogenesis, or epithelial barrier failure. Whether tumor type-specific bacterial species or the microbiome features reported by Nejmen et al.^1^ contribute to the induction of the indicated tumors is not clear. Further, tumoral bacteria have the ability to shape the tumor immune microenvironment, which can influence the efficacy of immunotherapy, and have also been suggested to strongly interact with drugs, influencing drug sensitivity.^5^ Nejmen et al.^1^ show that microbiome features are associated with the immune response in melanoma, while the correlation between the microbiome and the efficacy of cytotoxic drugs or targeted therapies needs further exploration. It is possible that treatment efficacy may be improved by manipulating the tumor microbiome.

Third, if the tumor microbiome influences tumor behavior and treatment responses, further dissection of how the intricate web of tumor cells, the microbiome, immune cells, and other possible environmental components cause this is needed at the cellular and molecular level. An unexpected finding by Nejmen et al.^1^ is that bacteria reside mainly in the cytoplasms of tumor and immune cells. Intriguingly, such bacteria are metabolically active, so it is possible that intracellular bacteria influence biological processes, particularly those related to cancer hallmarks, via their autonomous metabolism. Alternatively, does the microbiome shape the tumor microenvironment via a non-cell-autonomous mechanism? If so, what molecules mediate such effects? Answers to these questions may facilitate the development of microbiome-targeted treatments and improve the efficacies of current cancer treatments.

In summary, the comprehensive analysis of the tumor microbiome by Nejmen et al.^1^ is an essential first step toward deeper understanding of the influence of the tumor microbiome on the behaviors and responses of tumors to systemic therapies, which are a field of new opportunity in cancer biology. Future clinical studies considering these aspects are warranted and may help to improve the clinical efficacies of therapies.
